# Gastrointestinal Parasites Affecting Non-Human Primates That Are Kept Ex Situ and Their Handlers in Different Brazilian Institutions: Diagnosis and Analysis of Risk Factors

**DOI:** 10.3390/pathogens12121410

**Published:** 2023-11-30

**Authors:** Laís Verdan Dib, Alynne Barbosa, Breno da Silva, Alcides Pissinatti, Silvia Moreira, Maria Clotilde Tavares, Rodrigo Teixeira, André Luíz da Costa, José Augusto Muniz, Amauri Junglos, Zelinda Maria Hirano, Maria Regina Amendoeira

**Affiliations:** 1 Oswaldo Cruz Institute, Oswaldo Cruz Foundation (FIOCRUZ), Rio de Janeiro 21040-360, Brazil; alynnebarbosa@id.uff.br (A.B.); amendoeira.fiocruz@gmail.com (M.R.A.); 2 Medicine Faculty of Campos (FMC), Campos dos Goytacazes 28035-581, Brazil; 3 Department of Parasitology and Microbiology, Fluminense Federal University, Niterói 24020-140, Brazil; brenotorres@id.uff.br; 4 Primatology Center of Rio de Janeiro (CPRJ), Instituto Estadual do Ambiente (INEA), Guapimirim 25940-000, Brazil; alcidespissinatti@gmail.com (A.P.); silviabm.inea@gmail.com (S.M.); 5 Primatology Center, University of Brasília, Brasília 71750-000, Brazil; mchtavares@gmail.com; 6 Quinzinho de Barros Municipal Zoological Park (Zoo Sorocaba), Sorocaba 18020-268, Brazil; rhftzoo@hotmail.com (R.T.); almotacosta@yahoo.com.br (A.L.d.C.); 7 Wild Animals Graduate Program, Faculty of Veterinary Medicine and Zootechnics, Paulista University “Julio de Mesquita Filho” (UNESP-Botucatu), Botucatu 18618-970, Brazil; 8 Faculty of Veterinary Medicine, University of Sorocaba (UNISO), Sorocaba 18023-000, Brazil; 9 National Primate Center (CENP), Ananindeua 67033-009, Brazil; japcmuniz@gmail.com (J.A.M.); amauri.junglos@cenp.gov.br (A.J.); 10 Howler Project, Indaial 89086-000, Brazil; zehirano@hotmail.com

**Keywords:** non-human primates, protozoa, helminths, zoonosis, risk factors

## Abstract

Aim: Determining gastrointestinal parasites’ frequency in non-human primates (NHPs) and handlers in different Brazilian institutions, and associate it with management information. Methods: Different institutions in São Paulo (A), Brasília (B), Rio de Janeiro (C), Pará (D) and Santa Catarina (E) were included in the study. Fecal samples were processed by using coproparasitological techniques; information about NHP handling and professionals’ routine were collected through the use of questionnaires. Results: In total, 33.1% of 511 NHP samples were positive for parasites; the Entamoebidae Group and *Strongyloides* sp.-compatible eggs were the most detected protozoa and helminths, respectively. The Entamoebidae Group was mainly diagnosed in *Alouatta* from Institutions E and D, and was also the only parasite detected in handlers. *Strongyloides*-compatible eggs were mostly evident in fecal samples collected from Cebidae from Institutions B and D. Some risk factors associated with parasite infection were a high number of animals in the same enclosure and their use for research protocol purposes, whereas quarantine, a post-infection vacant period in enclosures and antiparasitic supply were categorized as protective factors against these agents’ infection. Conclusions: Parasites showing different transmission routes but concomitantly circulating in NHP institutions located in different Brazilian regions were identified in the current study, with an emphasis on those presenting zoonotic potential.

## 1. Introduction

Approximately 60% of all non-human primate (NHP) species worldwide are at some extinction risk level due to natural habitat reduction caused by the expansion of agricultural frontiers, logging and wild animal hunting, among others. Thus, the Ministry of the Environment, the Brazilian Institute for the Environment and Renewable Natural Resources (IBAMA) and Chico Mendes Institute for Biodiversity Conservation (ICMBio), together with other institutions, have been trying to develop strategies to guarantee NHP preservation and to reduce the impacts leading to biodiversity loss. One of the aforementioned strategies lies in the breeding and management processes for wild animals, which are carried out under human supervision [1]. In addition to conservation, wild animals can also be kept in institutions aimed at conducting biomedical research [2]. Overcrowding cases, poor hygiene standards, intensive management practices and poor feeding can trigger stress in animals kept under ex situ conditions. Consequently, this can weaken their immune system and make them more susceptible to developing parasitic infections [2,3]. It is important to emphasize that the phylogenetic proximity between humans and NHPs, as well as the long ex situ maintenance periods simians are subjected to, are factors capable of changing NHPs’ parasitological fauna and making them susceptible to infections caused by parasites that can determine zoonotic transmission cycles [2,4,5].

Thus, it is worth mentioning that parasitic diseases that can cause different clinical conditions, such as watery diarrhea, dysentery, colic, anorexia, and even death, are the main findings observed during NHP necropsy [6,7]. Gastrointestinal protozoa stand out among simian infections. *Balantioides coli* is the most frequent zoonotic parasite diagnosed in NHPs and several species are potential reservoir hosts to this protozoan [8]. *B. coli* infections in primates who are kept under human care have been detected in several zoos, such as ones in Belgium and Spain [6,9]. 

In addition to *B. coli*, the *Entamoeba histolytica*/*Entamoeba dispar* complex was also diagnosed in animals kept in zoos in France, Belgium, the United Kingdom, Brazil and Spain [5,6,10,11,12]. It is noteworthy that *Entamoeba histolytica* can lead to abdominal pain, apathy, lethargy, weakness, dehydration, gradual weight loss, anorexia, vomiting and severe catarrhal or hemorrhagic diarrhea, as well as to death in human and non-human primates. Some studies reported these protozoa in fecal samples collected from NHPs kept in different Brazilian institutions [5,13].

In addition to protozoa, helminths have also been widely diagnosed in non-human primates kept ex situ. Infections by strongyles, *Trichuris* sp., oxyurids and acanthocephalans were some of the most frequent findings in several countries such as Brazil, Ecuador and France [13,14,15,16,17,18,19,20]. Among strongyles, many species of *Strongyloides* can infect both New World and Old World primates and cause diarrhea, prostration, dyspnea, appetite loss and dehydration [21]. Infections by *Trichuris* sp. are very common and generally asymptomatic in Old World primates [22], while oxyurids can cause mild symptoms such as anal itching in NHPs [23]. Acanthocephalans, in turn, have been reported mainly among callitrichids, which present high rates of morbidity and mortality due to intestinal obstruction [24].

Although coproparasitological studies were conducted with NHPs kept under human care in different Brazilian states, they were limited to only one or few locations [5,13,14,15,16,17,25]. Furthermore, most reports presented a small sampling with a low species diversity [14,15,16,17,26,27,28,29]. Moreover, these studies did not apply a detailed statistical analysis by comparing the management procedures that the animals were subjected to and their parasites. This fact determines the shortage of information on this topic. In light of the foregoing, this study aimed to determine the frequency of gastrointestinal parasites in non-human primates and their handlers in five Brazilian institutions, based on associating parasite taxa with management information.

## 2. Materials and Methods

### 2.1. Sample Collection Sites and Ethical Aspects

**Figure 1:**
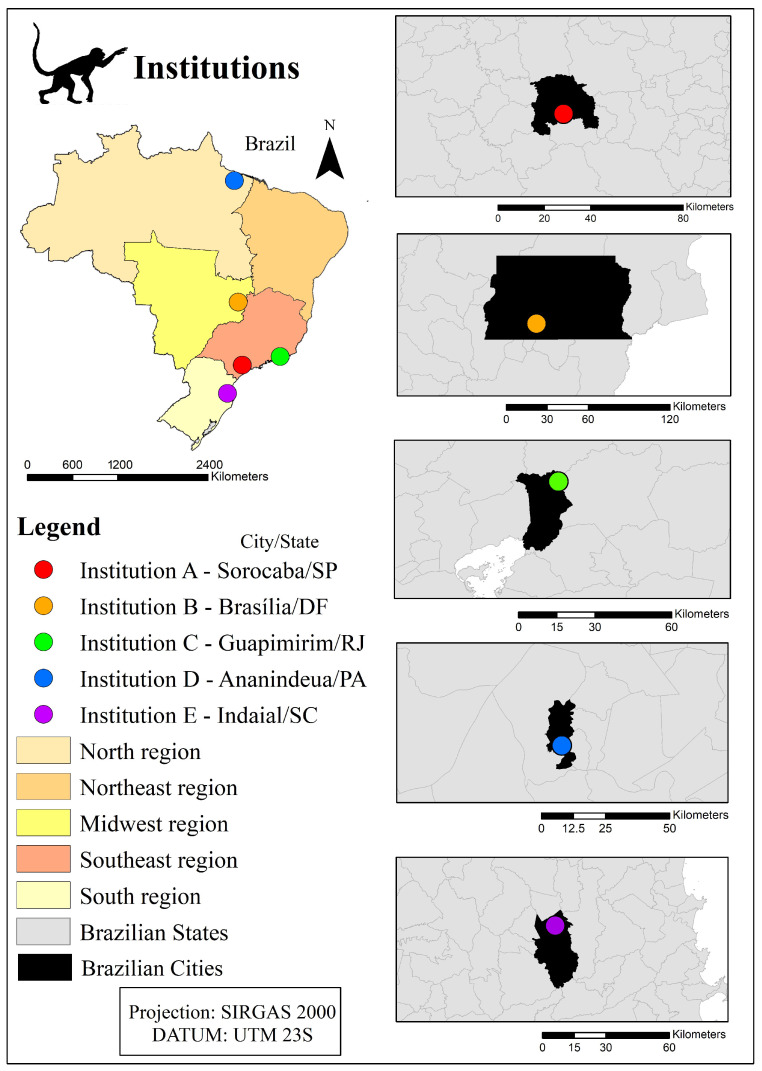
Location of the institutions (A–E) keeping non-human primates ex situ in different Brazilian states and regions. Source: developed by the authors.

Fecal samples from non-human primates kept ex situ, as well as from handlers, were collected in five institutions (A–E) located in different Brazilian regions (Figure 1). The present study was approved by the Biodiversity Authorization and Information System (SISBio), under license n. 74420–6 and authentication code 0744200620211025. It was also necessary to submit it to the Ethics Committee on Animal Research (also known as CEUA) of each participant institution. Institutions A, B and C were approved by Fiocruz’s CEUA, which decided to exempt the current study from the need of obtaining a license through the Brazilian Guideline for Animals’ Care and Use in Teaching or Scientific Research Activities, DBCA (Normative Resolution n. 30, CONCEA, 2016), item 6.1.10. Institution D was approved by CEUA/CENP/Evandro Chagas under registration n. 18/2020 and certificate n. 40/2021. Institution E, in its turn, was approved by CEUA from the Institution/Regional University of Blumenau on 23 February 2022. Furthermore, the present study was approved by the Human Ethics Committee of the Oswaldo Cruz Institute under opinion number 4.484.952 and CAAE 39957820.6.0000.5248.

### 2.2. Collecting Fecal Samples from Non-Human Primates and Handlers

Fecal samples from NHPs were collected straight from their enclosures’ floor by their handlers during the cleaning routine in order to minimize any stress or discomfort to the animals. In most cases, one sample per enclosure was collected. Given the small fecal volume produced by callitrichids and *Aotus*, these samples were collected in triplicate, i.e., they were collected from each enclosure once a day for three different and sequential days; this was carried out to get enough material to implement all the laboratory techniques.

Fecal material from animals who had recently arrived at the institutions as well as additional samples in the same enclosure, upon request by the institutions, were also collected out and included in the sampling. Moreover, feces from free-living animals found in Institutions A and B, who were in the surroundings of the enclosures, were also collected. Each group of fecal propagules found on the floor was counted as one fecal sample. Thus, the number of fecal samples collected from free-living NHPs does not necessarily correspond to the number of individuals living at Institutions A and B.

Data on New World, Old World, Great Ape and Prosimian NHP taxa, as well as on the number of enclosures, assessed individuals and collected samples, are shown in Supplementary Table S1. All fecal samples were stored in plastic bags without chemical preservatives and kept under refrigeration at 4 °C for three days maximum for laboratory analysis.

The handlers received a fecal-collection kit comprising two fecal collectors without chemical preservatives, two wooden sticks (115 mm × 9 mm) and instructions to properly collect and store fecal samples. After the kits were delivered to all participants, they were verbally instructed to collect fecal samples for two consecutive days. In addition, fecal samples collected on each day should be stored in individual sterile stool collectors and identified with the following information: handler’s name, collection date and time. All human samples were also kept under refrigeration at 4 °C for three days maximum.

### 2.3. Information Extracted from Questionnaires

Two epidemiological questionnaires were applied to professionals of the institutions. The first one comprised questions about their habits during the process of handling both the NHPs and their enclosures, the use of antiparasitic drugs, performed fecal examinations and gastrointestinal symptoms presented by these professionals. Participants were classified as primate handlers or food handlers. The first group comprised individuals who performed activities associated with the sanitary management of the enclosures and with the animals’ handling, or professionals who, besides accounting for the aforementioned functions, also carried out activities associated with feeding these NHPs. Food handlers, in their turn, were the ones who only performed tasks associated with feeding the animals, without having direct contact with the animals.

In addition, a second questionnaire was applied to the technical manager of each institution to collect information about each NHP colony. This questionnaire comprised questions about the frequency and type of food and drink provided to the animals, as well as about the performance of coproparasitological examinations, antiparasitic drug delivery to the animals and the therapeutic conduct adopted in case of infected and/or sick animals. This second questionnaire referred to all sampled enclosures; it did not include the free-living NHPs found in the institutions and the ones hospitalized in the local veterinary clinic.

### 2.4. Qualitative and Quantitative Coproparasitological Techniques

The direct examination technique was initially performed to investigate protozoan trophozoites right after all fresh NHP feces were collected and human samples were received. This initial analysis was carried out at the primate institutions. Subsequently, all samples were sent to the Parasitology Laboratory at Fluminense Federal University, where techniques, such as centrifuge-flotation with zinc sulfate solution (d = 1.180 g/mL) [30], modified centrifuge-sedimentation with ethyl [31,32] and spontaneous sedimentation were performed [33]. In cases of triplicate sampling, all three feces were individually analyzed through direct examination immediately after each collection. For the other parasitological techniques, the samples were processed as one pool of three samples. Microscopy slides’ reading, as well as parasite-shape morphometry and photomicrography, were carried out in Olympus BX 41 optical microscope coupled to a BEL^®^ EU12CONVS digital camera (Newcastle upon Tyne, UK).

Stool samples collected from NHPs who presented helminth eggs in, at least, one qualitative coproparasitological technique, and which had more than 4 g, were also subjected to Mini-FLOTAC technique with solutions comprising sodium chloride flotation (d = 1.200 g/mL) and zinc sulfate (d = 1.200 g/mL) in order to count the number of eggs per gram of feces (EPG). Two grams of feces were homogenized in 18 mL of solution in Fill-FLOTAC type-2 device, and they were used in each analysis. Then, 1 mL solution was added to each chamber for optical microscope reading purposes. At the end of the counting process, the value recorded for the two chambers was multiplied by the correction factor in order to estimate the number of EPG [34].

### 2.5. Data Analysis

Each triplicate sampling was counted as one sample. Fecal samples were classified as positive when at least one potentially infective parasitic form (trophozoite, cyst, larva or egg) was detected. The parasite prevalence was estimated by dividing the number of positive fecal samples by the total number of samples collected from each institution and also based on the NHPs’ taxonomic family. Chi-square test was carried out in Epi-Info software at 5% significance level. This was carried out to analyze the relevance of positivity based on both collection site and the NHPs’ taxonomic family.

The following variables were analyzed in the herein applied questionnaires: primates’ occupational density in each enclosure, use of animals for research protocols, animal feeding, food cleaning, animals’ water source, quarantine, enclosure floor, animals presenting gastrointestinal disorder symptoms in the previous six months, periodicity of antiparasitic drugs supplied to animals, coproparasitological examinations performed on animals, procedures applied to animals who tested positive for gastrointestinal parasites, enclosures’ hygienization, and post-infection vacant period in enclosures adopted in the investigated institutions.

Animals’ diet was defined as natural when it only comprised fruits and vegetables. Supplementation with other food sources took place when animals also received industrialized feed, as well as other food types, such as eggs, meat, chicken, bread, milk and insects. Item “Treatment applied to individuals and other procedures”, in variable “Procedures applied to animals who tested positive for gastrointestinal parasites”, refers to the treatment applied to parasitized individuals, as well as to animals in the enclosure and/or subjected to parasitological analysis to certify the parasitological cure for them. Variable “Enclosures’ hygienization” was classified into two different types: dry cleaning, which only comprised fecal and food residues’ sweeping and wet cleaning, which used water and chemical products (detergents and disinfectants) to wash the enclosures.

Univariate Chi-square or Fisher’s exact analysis was applied to the global positivity of gastrointestinal parasites and to each parasite taxon, in separate, at 5% significance level, after the variables addressed in the questionnaires were classified. Then, multivariate analysis based on logistic regression was applied to all variables showing statistical significance (*p* ≤ 0.05), at 5% significance level, for overall positivity of gastrointestinal parasites.

Free-living animals at Institutions A and B were not included in the uni- and multivariate analyses, since they were not housed in enclosures. Finally, Chi-square and Fisher’s exact tests were used to investigate the correlation between parasites’ frequency in samples collected from primates and food handlers, based on data extracted from questionnaires focused on these professionals’ routines in the institutions.

## 3. Results

### 3.1. Overall Gastrointestinal Parasites Positivity in Ex Situ Non-Human Primates

#### 3.1.1. Protozoa and Helminths’ Frequency among Brazilian Institutions

In total, 511 fecal samples from NHPs kept ex situ were collected at institutions located in different Brazilian regions. The overall positivity for potentially infective protozoa and helminths was 169 (33.1%). Protozoa (20.7%) were more often detected than helminths (13.7%), with a statistically significant difference (*p* < 0.05) among the institutions. Institution D was the one presenting the biggest sampling and, proportionally, the largest number of positive fecal samples, highlighting the diagnosis of protozoa. On the other hand, Institution C recorded the second-largest sampling, with a higher frequency of helminths (Table 1).

**Figure 2:**
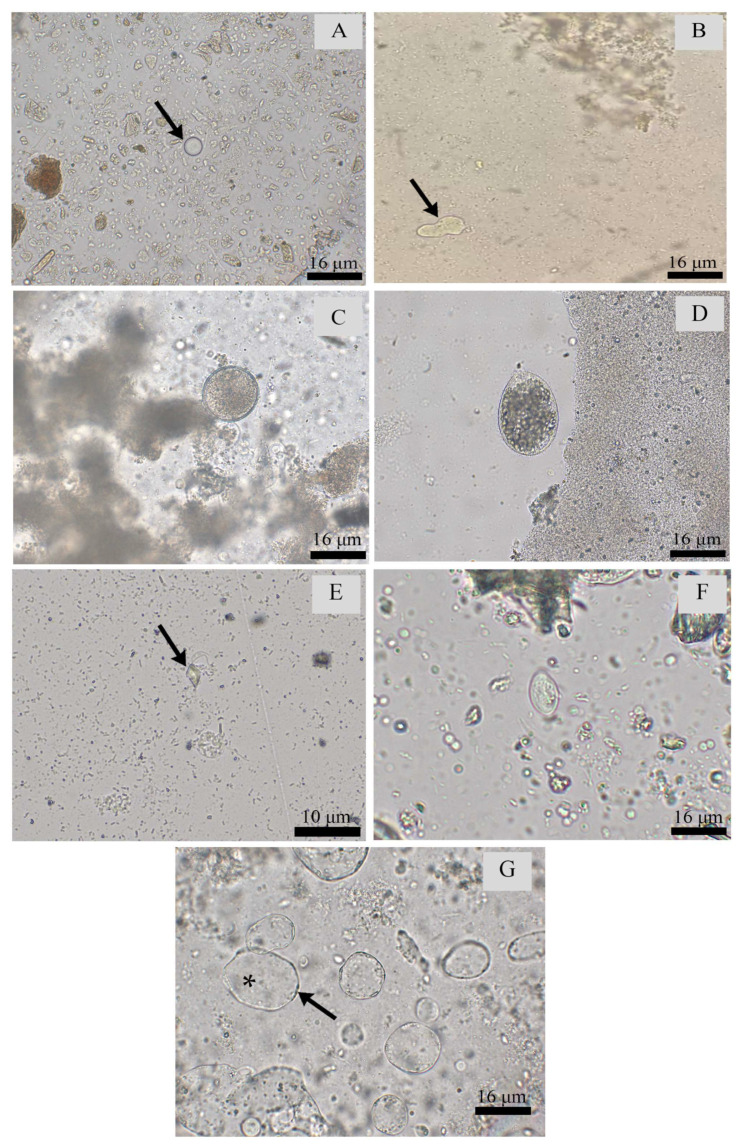
Protozoa detected in fecal samples from non-human primates of different institutions in Brazil. (**A**) Entamoebidae Group cyst (400×) (black arrow). (**B**) Entamoebidae Group trophozoite (400×) (black arrow). (**C**) Ciliophora Group cyst (400×). (**D**) Ciliophora Group trophozoite (400×). (**E**) Parabasalia Group trophozoite (1000×) (black arrow). (**F**) *Giardia duodenalis* cyst (400×). (**G**) *Blastocystis* sp. vacuolar form (*) with visible nuclei at the periphery (400×).

**Figure 3:**
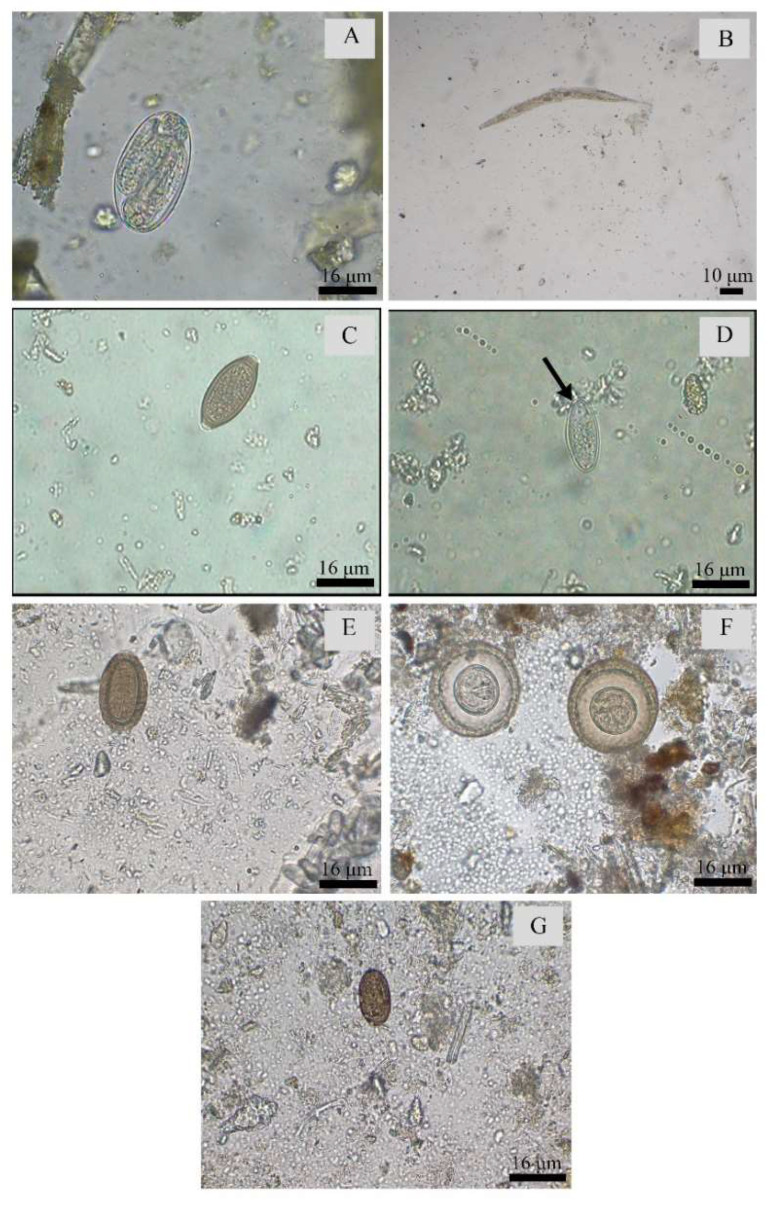
Helminths detected in fecal samples of non-human primates from different institutions in Brazil. (**A**) Thin-shelled nematode eggs (400×). (**B**) Nematode larvae (100×). (**C**) *Trichuris* sp. egg (400×). (**D**) Family Oxyuridae egg (400×) (black arrow). (**E**) Phylum Acanthocephala egg (400×). (**F**) *Hymenolepis* sp. eggs (400×). (**G**) Infraclass Trematoda egg (400×).

The Entamoebidae Group (cysts 5–12 µm ± 1.50 × 5–12 µm ± 1.28 and trophozoites) and Parabasalia (trophozoites) were the most detected parasitic forms (Figure 2). The Entamoebidae Group was diagnosed in all Institutions (Table 1). Among helminths, thin-shelled nematode eggs (62.9–111 µm ± 8.22 × 44.4–92.5 µm ± 6.11) and Acanthocephala eggs (111–240 µm ± 25.32 × 96.2–222 µm ± 25.96) were the two most detected (Figure 3). Both parasites were detected in Institutions B, C and D, although the former was also detected in Institution A (Table 1).

#### 3.1.2. Protozoa and Helminths’ Frequency among Ex Situ Non-Human Primates

Fecal samples from a wide variety of simians have been analyzed, including New World primates (Callitrichidae, Aotidae, Cebidae, Pitheciidae and Atelidae), Old World primates (Cercopithecidae and Hominidae) and Prosimians (Lemuridae). In general, the difference in parasite positivity among the primate families was statistically significant (*p* < 0.05) in almost all cases (Table 2).

Among the protozoa, the Entamoebidae Group was mostly detected in Atelidae, highlighting *Alouatta caraya* (28%) from Institution D and *Alouatta guariba* (24%) from Institution E, which accounted for approximately 25% of all samples that were positive for amoebas. Parasites of Phylum Ciliophora (cysts 51.8–59.2 µm ± 3.02 × 48.1–59.2 µm ± 4.65 and trophozoites) compatible with *Balantioides coli* were mainly diagnosed in *Alouatta guariba* (14.3%) from Institution C, *Pan troglodytes* (12.5%) from Institution A and *Sapajus libidinosus* (7.1%) from Institution B (Table 2, Table 3, Table 4, Table 5, Table 6 and Table 7). On the other hand, *Giardia duodenalis* (cysts 5–10 µm ± 2.16 × 3–4 µm ± 0.54) and *Blastocystis* sp. (vacuolar form) were the least detected protozoa (Table 1 and Figure 2).

**Figure 4:**
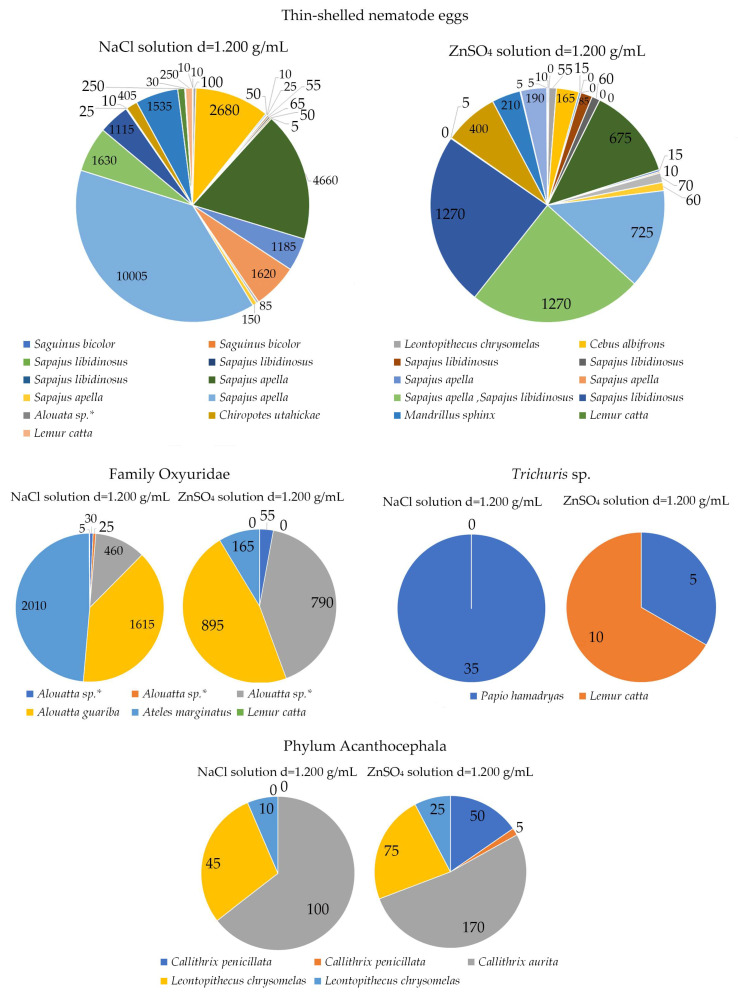
Helminth egg count per gram of feces (EPG) with two flotation solutions applied to Mini-FLOTAC in fecal samples collected from non-human primates kept in different Brazilian institutions. *: Free-living animals.

Thin-shelled nematode eggs were mainly detected in Cebidae (21.9%), whose samples also recorded the highest EPG rates, determined by using sodium chloride solution in the Mini-FLOTAC technique (Figure 4). Other helminth eggs were also diagnosed, such as Phylum Acanthocephala in *Callithrix*, *Saguinus* and *Leontopithecus,* as well as Oxyuridae (51.8–85.1 µm ± 7.57 × −26.9–48.1 µm ± 4.45) in Atelidae from Institution A. *Trichuris* sp. (55.5–88.8 µm ± 8.13 × 37–44.4 µm ± 2.69) eggs, in turn, were only detected in *Papio hamadryas* (25%) from Institution A and *Chlorocebus aethiops* (9.1%) from Institution D.

Among the Platyhelminthes, *Hymenolepis* sp. (27–35 µm ± 2.28 × 22–33 µm ± 11.72) eggs, without mamelon projections, were mainly detected in *Alouatta guariba* (14.3%) at Institution C. Infraclass Trematoda eggs (37–44.4 µm ± 3.09 × 18.5–25.9 µm ± 3, 09), which were compatible with *Platynosomum*, were diagnosed in *Chiropotes satanas* in Institution C, as well as in *Aotus infulatus* (2.1%) and *Chiropotes utahickae* in Institution D (50%) (Table 2, Table 3, Table 4, Table 5, Table 6 and Table 7 and Figure 3). Both *Hymenolepis* sp. and trematode eggs were also observed in the quantitative techniques. In *Alouatta guariba*, a total of 210 *Hymenolepis* sp. eggs were detected with a NaCl solution, and 185 eggs were diagnosed with a ZnSO_4_ solution. The sodium chloride solution was also more efficient at diagnosing trematode eggs in *Chiropotes Satanas*, whose EPG was 115 in comparison to the 35 eggs identified with a zinc sulfate solution (Figure 4).

Mixed infections in the same sample were observed in many primates at all the institutions. At Institution A, the most diverse case of co-infection occurred in *Lemur catta,* with one sample being positive for thin-shelled nematode eggs, larvae, *Trichuris* sp. and oxyurid eggs (Table 3). In addition to that, the same polyparasitisms were observed both in *Sapajus libidinosus* from Institution B and in *Saguinus bicolor* from Institution D (one sample with thin-shelled nematode eggs + nematode larvae and another with thin-shelled nematode eggs + acanthocephalans). At Institution E, Entamoebidae, Parabasalia, *Blastocystis* sp., and larvae were diagnosed in one sample of *Alouatta guariba* and a co-infection by Entamoebidae and Parabasalia in two other feces (Table 4 and Table 6). A similar scenario was evidenced in *A. guariba* at Institution C, which presented three samples with a great diversity of parasitic taxa (*Blastocystis* sp. + Parabasalia; Nematode larvae + Oxyuridae + Entamoebidae; and *Hymenolepis* sp. + *Giardia duodenalis* + Parabasalia + Ciliophora + Entamoebidae) (Table 5). At Institution D, some other cases of polyparasitism were observed in one sample of hybrid callitrichids (Ciliophora *+ G. duodenalis*); in two feces of *A. infulatus* (one with Entamoebidae + *Blastocystis* sp. and the other positive for thin-shelled nematode eggs + trematodes); in *Saimiri collinsi* (two samples with Parabasalia + Entamoebidae and one with thin-shelled nematode eggs + nematode larvae); and in *Chlorocebus aethiops* (one sample with Entamoebidae + *Blastocystis* sp.; another one with Entamoebidae + *Trichuris* sp.; and two feces positive for *Giardia duodenalis* + Entamoebidae) (Table 6 and Table 7).

In addition to animals kept ex situ in enclosures, three taxa of free-living animals were observed: an *Alouatta* group at Institution A and *Callithrix* and *Sapajus* groups at Institution B. In those cases, Oxyuridae eggs were found in 15% of the *Alouatta* samples, and 80% of the feces from *Callithrix* were positive for Phylum Acanthocephala eggs (Table 3 and Table 4; Figure 3).

#### 3.1.3. Risks and Protective Factors Associated with Gastrointestinal Parasite’s Positivity in NHPs

The correlation between the management information collected through questionnaires and parasites’ positivity had a significant association (*p* < 0.05) in univariate analysis based on Chi-square or Fisher’s exact test (Supplementary Table S2). However, only some of them remained statistically significant when they were compared, all together, through multivariate logistic regression analysis (Table 8 and Table 9). In general, the animals’ occupational density in the enclosures and the use of animals for research protocol purposes were considered risk factors that favored the infection by parasites in NHPs, whereas quarantine, the supply of antiparasitic agents and the adoption of post-infection vacant period in enclosures were associated with protective factors (Table 8 and Table 9).

In relation to each parasitic taxon when detected individually, the enclosures’ hygienization type played a relevant role against infections caused by protozoa belonging to the Entamoebidae Group. NHPs kept in enclosures where dry and wet cleaning was carried out together were 4.45 times more likely to be parasitized by this protozoa group than in enclosures that were only submitted to dry cleaning (fecal and food waste sweeping) (Table 9). Infection by the Parabasalia Group was associated with animals’ use for research protocols. In this case, asymptomatic NHPs were more likely to be infected by parabasilids than the symptomatic ones (Table 8 and Table 9).

Several activities associated with animal handling were identified as protective factors. In other words, they contributed to reducing the parasitic infection rates. The following measures were associated with a lower frequency of nematode eggs in NHPs: cleaning food with water and chemical products, quarantine and use of antiparasitic drugs (Table 8 and Table 9). In relation to Phylum Acanthocephala, only enclosures’ hygienization by using wet cleaning (water and other chemical products) was statistically relevant in terms of helping to control this parasitic infection (Table 9). In addition to that, the variable “source of water provided to the animals” (treated water used for public supply) was associated with favoring infections by nematode larvae (Table 8).

#### 3.1.4. Gastrointestinal parasites in handlers and potential risk factors

Only cystic forms of the Entamoebidae Group were evidenced in 16.2% of handlers’ fecal samples. A statistically significant difference (*p* < 0.05) was observed in the amoebid frequency among all five institutions, especially in professionals from Institution D, which is located in northern Brazil (Table 10 and Table 11).

## 4. Discussion

The overall positivity for potentially infective protozoa and helminths in NHPs from five Brazilian institutions was 33.1%. Positivity rates lower than the ones recorded in the current study were reported in fecal samples from primates kept in zoos in the United Kingdom, China, and in Maranhão State, Brazil [10,14,24]. However, higher parasitic frequencies were evidenced in most studies, highlighting the coproparasitological surveys of NHPs kept ex situ in Brazil, France, Kenya, Malaysia and Bangladesh [5,13,15,19,27,29,35].

It is noteworthy that the parasite rates in this study may be associated with a different climate, the institution’s location, the NHP species diversity, their behavioral habits and the primate’s susceptibility to parasitic infections. In addition, the methodologies used to collect fecal samples in this study, the variety of laboratory techniques, as well as the goals of each participating institution, may have influenced the results obtained in this survey. In this study, a single fecal collection of the entire primate colony was carried out, so that it was possible to have an overview of the parasites in NHPs from institutions spread all over Brazil. However, the analysis of only one sample may have underestimated the positivity in this survey, since the parasites are intermittently released in feces.

Protozoa were more often detected in NHPs’ feces than helminths. A similar scenario was reported in biomedical institutions in Rio de Janeiro, Brazil, as well as in zoos in Bangladesh, Belgium, Nigeria and France [5,6,19,36,37]. The highest protozoa frequency may have been favored by these agents’ direct biological cycle, low infective dose, high cyst resistance in the environment and short pre-patent period and by the cysts’ infective capacity right after their excretion [5,13,36]. In addition, protozoa have a low sensitivity to anthelmintics, which are often used as the main prophylactic antiparasitic protocol. Added to that, the high prevalence of protozoa in feces from Cercopithecidae and Hominidae may have increased its global rate, since these simians are more susceptible [5].

Most of the fecal samples that were positive for parasites are from Institutions D and E, respectively. The Entamoebidae Group stood out, since it was detected in all the institutions and in a great variety of species. However, a predominance of amoebas was observed in *Alouatta* when comparing with other primates. Thus, it is possible that the susceptibility of howler monkeys may have favored the higher positivity for the Entamoebidae Group in Institution E, since this place is exclusively used to breed and conserve *Alouatta guariba* (red howler monkey). It is worth mentioning that the diagnosis of Entamoebidae Group must be analyzed with caution, since reports of *Alouatta* individuals’ deaths by *Entamoeba histolytica* have already been reported in Brazil [38].

It is well known that both NHPs and humans can be infected by *E. histolytica*, *E. dispar*, *E. hartmanni*, *E. nuttalli*, *E. polecki*, *E. chattoni*, *E. suis* and *E. coli*, a fact that increases the possibility of zoonotic transmissions [39]. Similar to a human infection, *E. histolytica* can lead to severe symptomatic clinical cases of amoebiasis in non-human primates [39,40,41]. Only amoebas were detected in fecal samples from handlers in the current study. Most of the infected professionals work at Institution D, which is located in Pará, a northern Brazilian state. This great frequency was already expected, since this Brazilian state accounts for the highest national rates of human infections caused by *Entamoeba histolytica* due to a lack of basic sanitation, which acts in association with its humid tropical climate [42,43].

Although there was no statistically significant association among handler’s parasitological results and their individual information and activities at the institutions, it is important to call attention to the diagnosis of the Entamoebidae Group in their samples. This result highlights the importance of intensifying the adoption of prophylactic measures, especially on periodic parasitological examinations and mediation of information about parasites with zoonotic potential at the institutions.

Trophozoites and cysts of Phylum Ciliophora (morphologically compatible with *Balantioides coli*), Parabasalia Group trophozoites, cysts of *Giardia duodenalis* and vacuolar forms of *Blastocystis* sp. were also detected in the feces of NHPs. All these taxa were mainly detected in neotropical primates and were concomitantly identified in Institutions C and D. In addition, prevalent diagnoses of Parabasalia Group and *G*. *duodenalis* in the primate’s taxonomic family were observed, since both of them were not detected in Pitheciidae, Cercopithecidae and Lemuridae, and *G. duodenalis* was not detected in Aotidae, Cebidae and Hominidae. These protozoa are known to infect several species, even humans, highlighting their potential for zoonotic transmission. Still, some of them have already had their clinical relevance for NHPs confirmed, a fact that emphasizes the relevance of the parasitological monitoring carried out in the present study [12,44].

It is worth noting that although mainly protozoa were detected in this study, the positivity of some taxa may have been underestimated, such as *Blastocystis* sp. One of the reasons may have been the collection of fecal samples without chemical preservatives and the disruption of this protozoan due to some stages of laboratory processing being carried out with water [45].

*Balantioides coli* is reported to determine dysentery cases in baboons, as well as death cases in chimpanzees and gorillas. Moreover, it is pointed out as one of the main parasites capable of reducing the milk fat content in *Macaca mulatta*, a fact that impairs the development of lactating animals [46,47,48,49]. Although the real clinical relevance of *Giardia duodenalis*, Parabasalia Group and *Blastocystis* sp. for non-human primates remains unknown [12,50,51], some studies have pointed out some symptomatic diarrheal infections, mainly caused by *Giardia duodenalis*, in juvenile non-human primates [50,52].

Thin-shelled nematode eggs, oval-shaped, embryonated and sometimes with larva inside, were the most often detected helminths. These parasites were proportionally more diagnosed in Institutions B and D, mainly in *Sapajus*. Similar results were reported in NHPs kept ex situ in Ecuador and Brazil [15,16,17,20].

Eggs and larvae observed in the current study were not deeply classified based on morphological and molecular analyses. Nevertheless, their incidence in NHPs reinforces the possibility that they could be *Strongyloides cebus*, which is the only *Strongyloides* species described as infecting neo-tropical primates [20,53,54]. Furthermore, the possibility that the parasites identified could be other species of *Strongyloides* or even free-living nematodes cannot be ruled out. The herein investigated institutions are located in regions with different climates. Institution B is characterized by a tropical climate with dry winter (Aw), and Institution D has a tropical humid climate (Am) based on the Köppen–Geiger classification. Both presented favorable environmental conditions for infection persistence. The humid environment in Institution D, and part of the enclosure floors being composed of earth in Institution B, may have favored the development of parasites’ infective forms.

It is important to highlight that enclosures with a lack of environmental enrichment can stimulate animals to spend longer time moving on the ground, which increases the chances of penetration by infective larvae [20]. Although *Strongyloides* is often detected in NHPs’ feces, it is not possible to rule out the possibility that these thin-shelled eggs belong to genera *Ancylostoma*, *Necator* or *Trichostrongylus*, since their morphology is quite similar. However, regardless of the taxonomic classification, this finding deserves attention, since all the aforementioned genera comprise species capable of infecting humans.

Although this study did not aim to compare the flotation solutions used in the quantitative Mini-FLOTAC technique, it was observed that the sodium chloride solution obtained the highest recovery rates of parasitic structures, mainly thin-shelled nematode eggs. This result was already expected, as the greater sensitivity of detection of these eggs by this solution has already been reported [55].

In addition to thin-shelled nematode eggs, eggs that were compatible with *Trichuris trichiura* and Family Oxyuridae were also detected in this study. It is worth emphasizing that species *T. trichiura* was only identified in Old World primates, whose susceptibility to infection caused by this nematode was previously reported in other studies [5,9,13,15,24]. Moreover, the coproparasitological diagnosis of *T. trichiura* in Old World non-human primates ends up being recurrent due to the eggs’ high resistance in the environment and the difficulty in eliminating adult individuals that are deeply inserted into the cecal mucosa. Although the taxonomic classification of Family Oxyuridae has not yet been explored, neotropical animals are often infected by *Trypanoxyuris* [36,50]. Both nematodes can determine symptomatic infections with mucoid diarrhea in heavy infections of *T. trichiura*, as well as anal itching and irritation episodes in the case of pinworms [23,40].

Helminths showing indirect biological cycles were also diagnosed, highlighting the Phylum Acanthocephala eggs. They were mainly detected in Callitrichidae from Institution C. According to previous parasitological studies carried out in this same location, acanthocephalan eggs were detected in *Leontopithecus*, whereas adults of *Prosthernorchis elegans* were recovered in necropsies of *Callithrix aurita* and *Leontopithecus rosalia* [5,56]. It is worth mentioning that this parasite taxon can be lethal to primates, mainly to callitrichids, due to secondary bacterial infections resulting from nodules grown in the submucosa and intussusception, as well as from ileum and cecum obstruction [24,57]. In addition, there is no pharmacological treatment available on the market to rule out infection caused by *P. elegans* [24].

Eggs of *Hymenolepis*, morphologically compatible with *H. diminuta*, were only detected in *Alouatta guariba* and *Plecturocebus vieirai*. Also, trematode eggs similar to Dicrocoeliidae were identified in *Chiropotes* sp. and *Aotus infulatus*. Both parasites can determine clinical changes, such as diarrhea and/or weight loss [58,59]. Infections caused by *Hymenolepis* and Dicrocoelidae, most specifically by *Platynosomum illiciens*, have been previously reported in primates kept in Institutions C and D, respectively [5,60]. It is important to emphasize that the detection of the aforementioned parasite taxa in ex situ NHPs’ feces is overall uncommon, since primates end up eating parasitized intermediate or paratenic hosts, such as arthropods, reptiles and amphibians. Overall, open enclosures close to forest areas favor the entry of intermediate and paratenic hosts; consequently, they increase the possibility of infection caused by these parasites in NHPs. This scenario was clearly observed in Institution C, since the enclosures were built closer to the environment in order to increase animal welfare.

Based on the association between the parasitic frequency among institutions and information on the questionnaires, it was clear that a higher occupational density and the use of NHPs for biomedical-experiment-based research purposes have favored infections caused by helminths and protozoa. The primates’ agglomeration in enclosures favors fecal material concentration and parasites transmission, besides triggering animals’ stress and weakening their immunity [5,16]. In addition, intensive handling and animals’ participation in research protocols may be other immunocompromising factors [61]. In the latter case, it is important to consider that these infections can affect the findings of the research. This factor highlights the need to conduct studies like the present one, which conducted a full parasitological survey comprising all enclosures, including those intended for this purpose. On the other hand, some prophylactic measures taken by the institutions participating in the study—such as quarantine, post-infection vacant period in enclosures and antiparasitic drug administration to the animals—have played a key role in reducing the number of parasitic infections in NHPs.

The enclosures’ cleaning process, which mostly comprised dry organic material removal followed by washing with water, detergents and/or disinfectants, was another factor associated with gastrointestinal parasites’ prophylaxis. This cleaning procedure proved to be efficient in controlling helminthic infections by Phylum Acanthocephala, since it helped remove potential intermediate hosts. However, this hygienic measure was not efficient in controlling the transmission of Entamoebidae Group. Furthermore, social grooming and primates’ behavior to manipulate their anal region may have favored reinfections by this protozoan. Thus, the amoebas’ frequency may not have been directly influenced by environmental hygiene.

Both quarantine and antiparasitic drug administration were associated with a reduced frequency of nematode thin-shelled eggs. This finding was already expected, since most antiparasitic drugs provided to the investigated animals mainly act on nematodes [5,13]. This fact was corroborated in the present study, since most professionals responsible for providing care to the investigated animals reported to often administer ivermectin to NHPs as an antiparasitic agent.

Interestingly, non-human primates, even the asymptomatic ones, recorded higher chances of being infected by parabasilids. As previously emphasized, the real importance of this protozoa group for the health of non-human primates remains unknown. However, some studies have pointed out that they can be commensal organisms that cause no harm to these animals [62]. A lack of clinical changes appears to have favored the use of these animals for the biomedical-experiment-based research projects implemented in Institution D.

Only the treated water used for public supply was associated with an incidence of larval forms in animals’ feces. Although this finding seems worrisome, it must be analyzed with caution, since the samples were collected right from the enclosures’ floor, and the larvae may be from free-living nematodes. This issue was mainly observed in Institution A, which is located close to an urban center. Unfortunately, comparisons with other studies about the correlation between gastrointestinal parasites’ frequency and management procedures carried out in NHP institutions under human care were impaired, since none of the analyzed articles addressed this topic. Most of them only described protozoan and helminth frequencies and did not analyze risk factors associated with parasitological survey. Therefore, the current study has a pioneering nature, since it was the first study carried out in Brazil to extrapolate parasite positivity data, as well as to highlight positive management points that should be maintained in institution protocols, such as quarantine, a post-infection vacant period in enclosures and antiparasitic drug supply to animals at least once a year.

It is worth mentioning that although the present study has diagnosed different parasitic taxa in five Brazilian institutions, these positivity rates were lower than those observed in most parasitological studies carried out with ex situ NHPs. These findings resulted from good management practices applied to primates daily, as well as from the care provided by the institutions to both the facilities and the animals, despite their limited financial resources. Thus, the current parasitological survey helped us better understand the presence of gastrointestinal parasites in institutions used for different purposes and located in different Brazilian regions. Based on all the analyses, it is worth emphasizing the need for a parasitological diagnosis routine to enable prescribing proper treatments, mainly for protozoa infections. These procedures can help ex situ NHP conservation processes, as well as help avoiding zoonotic transmission and parasites’ resistance to antiparasitic drugs, both in non-human primates and in professionals who work directly with these animals.

## Figures and Tables

**Table 1:** Overall positivity of gastrointestinal parasites detected in fecal samples collected from ex situ non-human primates kept in different institutions (A, B, C, D and E) in Brazil, from March 2021 to June 2023.

Parasitic Taxa	A (n = 68)	B (n = 36)	C (n = 176)	D (n = 206)	E (n = 25)	Total (n = 511)	*p*-Value ª
Protozoa	8 (11.8%)	2 (5.5%)	12 (6.8%)	77 (37.4%)	7 (28%)	106 (20.7%)	0.000 *
Entamoebidae	7 (10.3%)	1 (2.8%)	7 (4%)	40 (19.4%)	6 (24%)	61 (11.9%)	0.000 *
Ciliophora	1 (1.5%)	1 (2.8%)	1 (0.6%)	3 (1.5%)	-	6 (1.2%)	0.764
Parabasalia	2 (2.9%)	-	7 (4%)	41 (19.9%)	4 (16%)	54 (10.6%)	0.000 *
*Giardia duodenalis*	-	-	1 (0.6%)	3 (1.5%)	-	4 (0.8%)	0.6762
*Blastocystis* sp.	-	-	1 (0.6%)	5 (2.4%)	1 (4%)	7 (1.4%)	0.2611
Helminths	14 (20.6%)	15 (41.7%)	19 (10.8%)	21 (10.2%)	1 (4%)	70 (13.7%)	0.000 *
Thin-shelled nematode eggs	6 (8.8%)	8 (22.2%)	1 (0.6%)	20 (9.7%)	-	35 (6.8%)	0.000 *
Nematode larvae	8 (11.8%)	6 (16.7%)	3 (1.7%)	2 (1%)	1 (4%)	20 (3.9%)	0.000 *
*Trichuris* sp.	2 (2.9%)	-	-	1 (0.5%)	-	3 (0.6%)	0.095
Oxyuridae	5 (7.4%)	-	1 (0.6%)	-	-	6 (1.2%)	0.000 *
Acanthocephala	-	7 (19.4%)	13 (7.4%)	1 (0.5%)	-	21 (4.1%)	0.000 *
*Hymenolepis* sp.	-	-	2 (1.1%)	-	-	2 (0.4%)	0.430
Trematoda	-	-	1 (0.6%)	2 (1%)	-	3 (0.6%)	0.864
Total (n = 511)	22 (32.3%)	16 (44.4%)	28 (15.9%)	96 (46.6%)	7 (28%)	169 (33.1%)	0.000 *

ª—Chi-square test * *p* < 0.05; “-”: zero; A, B, C, D, E: institutions; n: number of samples.

**Table 2:** Frequency of gastrointestinal parasites detected among different non-human primate families kept ex situ in Brazilian institutions, from March 2021 to June 2023.

Parasitic Taxa	Family								Total (n = 511)	*p*-Value ª
	Callitrichidae (n = 225)	Aotidae (n = 54)	Cebidae (n = 96)	Pitheciidae (n = 14)	Atelidae (n = 95)	Cercopithecidae (n = 16)	Hominidae (n = 8)	Lemuridae (n = 3)		
Protozoa	22 (9.8%)	9 (16.7%)	35 (36.4%)	2 (14.3%)	24 (25.3%)	7 (43.7%)	7 (87.5%)	-	106 (20.7%)	0.000 *
Entamoebidae	11 (4.9%)	13 (24.1%)	7 (7.3%)	2 (14.3%)	15 (15.8%)	7 (43.7%)	6 (75%)	-	61 (11.9%)	0.000 *
Ciliophora	2 (0.9%)	-	2 (2.1%)	-	1 (1%)	-	1 (12.5%)	-	6 (1.2%)	0.040 *
Parabasalia	11 (4.9%)	2 (3.7%)	28 (29.2%)	-	12 (12.6%)	-	1 (12.5%)	-	54 (10.6%)	0.000 *
*Giardia duodenalis*	1 (0.4%)	-	-	-	1 (1%)	2 (12.5%)	-	-	4 (0.8%)	0.000 *
*Blastocystis* sp.	-	2 (3.7%)	1 (1%)	-	3 (3.2%)	1 (6.2%)	-	-	7 (1.4%)	0.170
Helminths	27 (12%)	2 (3.7%)	22 (22.9%)	5 (35.7%)	8 (8.4%)	3 (18.7%)	-	3 (100%)	70 (13.7%)	0.000 *
Thin-shelled nematode eggs	4 (1.8%)	2 (3.7%)	21 (21.9%)	1 (7.1%)	3 (3.2%)	1 (6.2%)	-	3 (100%)	35 (6.8%)	0.000 *
Nematode larvae	7 (3.1%)	-	6 (6.2%)	1 (7.1%)	3 (3.2%)	-	-	3 (100%)	20 (3.9%)	0.000 *
*Trichuris* sp.	-	-	-	-	-	2 (12.5%)	-	1 (33.3%)	3 (0.6%)	0.000 *
Oxyuridae	-	-	-	-	5 (5.3%)	-	-	1 (33.3%)	6 (1.2%)	0.000 *
Acanthocephala	20 (8.9%)	-	1 (1%)	-	-	-	-	-	21 (4.1%)	0.001 *
*Hymenolepis* sp.	-	-	-	1 (7.1%)	1 (1%)	-	-	-	2 (0.4%)	0.008 *
Trematoda	-	1 (1.8%)	-	2 (14.3%)	-	-	-	-	3 (0.6%)	0.000 *
Total (n = 511)	48 (21.3%)	11 (20.4%)	54 (56.2%)	7 (50%)	30 (31.6%)	9 (56.2%)	7 (87.5%)	3 (100%)	169 (33.1%)	0.000 *

ª—Chi-square test; * *p* < 0.05; “-”: zero; n: number of samples

**Table 3:** Gastrointestinal parasites detected in fecal samples collected from non-human primates kept ex situ in Institution A, São Paulo State, southeastern Brazil.

Non-Human Primate Taxa	Entamoebidae	Ciliophora	Parabasalia	Thin-Shelled Nematode Egg	Nematode Larvae	*Trichuris* sp.	Family Oxyuridae	Total
A								
Callitrichidae								
*Saguinus fuscicollis* (n = 1)	-	-	-	-	1 (100%)	-	-	1 (100%)
*Saguinus midas* (n = 1)	-	-	-	-	1 (100%)	-	-	1 (100%)
*Leontopithecus rosalia* (n = 1)	-	-	-	-	1 (100%)	-	-	1 (100%)
Aotidae								
*Aotus trivirgatus* (n = 3)	1 (33.3%)	-	1 (33.3%)	-	-	-	-	1 (33.3%) **
Pitheciidae								
*Plecturocebus vieirai* (n = 3)	-	-	-	-	1 (33.3%)	-	-	1 (33.3%)
Atelidae								
*Alouatta* sp. (n = 20) *	-	-	-	2 (10%)	-	-	3 (15%)	3 (15%) **
*Ateles marginatus* (n = 6) **	-	-	-	-	1 (16.7%)	-	1 (16.7%)	2 (33.3%)
Cercopithecidae								
*Mandrillus sphinx* (n = 1)	-	-	-	1 (100%)	-	-	-	1 (100%)
*Papio hamadryas* (n = 4)	-	-	-	-	-	1 (25%)	-	1 (25%)
Hominidae								
*Pan troglodytes* (n = 8) **	6 (75%)	1 (12.5%)	1 (12.5%)	-	-	-	-	7 (87.5%)
Lemuridae								
*Lemur catta* (n = 3) **	-	-	-	3 (100%)	3 (100%)	1 (33.3%)	1 (33.3%)	3 (66.7%) **
Total (n = 68)	7 (10.3%)	1 (1.5%)	2 (2.9%)	6 (8.6%)	8 (11.8%)	2 (2.9%)	5 (7.3%)	22 (32.3%)

*: Free-living animals; **: Mixed infections; - n: number of samples.

**Table 4:** Gastrointestinal parasites detected in fecal samples collected from non-human primates kept ex situ in Institutions B and E, which are located in the Federal District, midwestern Brazil and in Santa Catarina State, southern Brazil, respectively.

Non-Human Primate Taxa	Entamoebidae	Ciliophora	Parabasalia	*Blastocystis* sp.	Thin-Shelled Nematode Egg	Nematode Larvae	Phylum Acanthocephala	Total
B								
Callitrichidae								
*Callithrix* sp. (n = 5) *	-	-	-	-	-	1 (20%)	4 (80%)	4 (80%) **
*Callithrix penicillata* (n = 15)	-	-	-	-	-	-	2 (13.3%)	2 (13.3%)
Cebidae								
*Sapajus* sp. (n = 2) *	-	-	-	-	2 (100%)	2 (100%)	-	2 (100%) **
*Sapajus libidinosus* (n = 14)	1 (7.1%)	1 (7.1%)	-	-	6 (42.8%)	3 (21.4%)	1 (7.1%)	10 (71.4%) **
Total (n = 36)	1 (2.8%)	1 (2.8%)	-	-	8 (22.2%)	6 (16.7%)	7 (19.4%)	16 (44.4%)
E								
Atelidae								
*Alouatta guariba* ** (n = 25)	6 (24%)	-	4 (16%)	1 (4%)	-	1 (4%)	-	7 (28%)

*: Free-living animals at the institution; **: mixed infections; n: number of samples.

**Table 5:** Gastrointestinal parasites detected in fecal samples collected from non-human primates kept ex situ in Institution C, Rio de Janeiro State, southeastern Brazil.

Non-Human Primate Taxa	Entamoebidae	Ciliophora	Parabasalia	*Giardia duodenalis*	*Blastocystis* sp.	Thin-Shelled Nematode Egg	Nematode Larvae	Family Oxyuridae	Phylum Acanthocephala	*Hymenolepis* sp.	Infraclass Trematoda	Total
C												
Callitrichidae												
*Callithrix aurita* (n = 16)	-	-	-	-	-	-	-	-	4 (25%)	-	-	4 (25%)
*Callithrix geoffroyi* (n = 1)	-	-	-	-	-	-	-	-	1 (100%)	-	-	1 (100%)
*Saguinus bicolor* (n = 5)	2 (40%)	-	-	-	-	-	-	-	-	-	-	2 (40%)
*Leontopithecus* *chrysomelas* (n = 93)	1 (1.1%)	-	2 (2.1%)	-	-	1 (1.1%)	2 (2.1%)	-	7 (7.5%)	-	-	11 (11.8%) **
*Leontopithecus chrysopygus* (n = 4)	-	-	1 (25%)	-	-	-	-	-	-	-	-	1 (25%)
*Leontopithecus rosalia* (n = 8)	-	-	-	-	-	-	-	-	1 (12.5%)	-	-	1 (12.5%)
Hybrid *Leontopithecus* (n = 1)	-	-	1 (100%)	-	-	-	-	-	-	-	-	1 (100%)
Cebidae												
*Sapajus xanthosternos* (n = 12)	1 (8.3%)	-	1 (8.3%)	-	-	-	-	-	-	-	-	1 (8.3%) **
Pitheciidae												
*Pithecia mittermeieri* (n = 1)	1 (100%)	-	-	-	-	-	-	-	-	-	-	1 (100%)
*Plecturocebus vieirai* (n = 1)	-	-	-	-	-	-	-	-	-	1 (100%)	-	1 (100%)
*Chiropotes satanas* (n = 1)	-	-	-	-	-	-	-	-	-	-	1 (100%)	1 (100%)
Atelidae												
*Alouatta caraya* (n = 1)	-	-	-	-	-	-	1 (100%)	-	-	-	-	1 (100%)
*Alouatta guariba* (n = 7)	2 (28.6%)	1 (14.3%)	2 (28.6%)	1 (14.3%)	1 (14.3%)	-	-	1 (14.3%)	-	1 (14.3%)	-	3 (42.8%) **
Total (n = 176)	7 (4%)	1 (0.6%)	7 (4%)	1 (0.6%)	1 (0.6%)	1 (0.6%)	3 (1.7%)	1 (0.6%)	13 (7.4%)	2 (1.1%)	1 (0.6%)	28 (15.9%)

**: Mixed infections; n: number of samples.

**Table 6:** Gastrointestinal parasites detected in fecal samples collected from non-human primates kept ex situ in Institution D, Pará State, northern Brazil.

Non-Human Primate Taxa	Entamoebidae	Ciliophora	Parabasalia	*Giardia* *duodenalis*	*Blastocystis* sp.	Thin-Shelled Nematode Egg	Nematode Larvae	*Trichuris* sp.	Phylum Acanthocephala	Infraclass Trematoda	Total
D											
Callitrichidae											
*Callithrix penicillata* (n = 2)	2 (100%)	-	-	-	-	-	-	-	-	-	2 (100%)
*Callithrix jacchus* (n = 6)	1 (16.7%)	-	-	-	-	-	-	-	-	-	1 (16.7%)
Hybrid *C. jacchus x C. penicillata* (n = 14)	-	1 (7.1%)	-	1 (7.1%)	-	1 (7.1%)	-	-	-	-	2 (14.3%) **
*Mico humeralifer* and *Leontopithecus chrysomelas* (n = 1)	1 (100%)	-	-	-	-	-	-	-	-	-	1 (100%)
*Saguinus ursulus* (n = 8)	1 (12.5%)	-	1 (12.5%)	-	-	-	-	-	-	-	2 (25%)
*Saguinus midas* and *Callicebus* sp. (n = 1)	1 (100%)	1 (100%)	-	-	-	-	-	-	-	-	1 (100%) **
*Saguinus bicolor* (n = 3)	-	-	-	-	-	2 (66.7%)	1 (33.3%)	-	1 (33.3%)	-	2 (66.7%) **
*Leontocebus weddelli* (n = 11)	1 (9.1%)	-	6 (54.5%)	-	-	-	-	-	-	-	6 (54.5%) **
*Callimico goeldii* (n = 4)	1 (25%)	-	-	-	-	-	-	-	-	-	1 (25%)
Aotidae											
*Aotus infulatus* (n = 48)	8 (16.7%)	-	-	-	1 (2.1%)	2 (4.2%)	-	-	-	1 (2.1%)	10 (20.8%) **

**: Mixed infections; n: number of samples.

**Table 7:** Gastrointestinal parasites detected in fecal samples collected from non-human primates kept ex situ in Institution D, Pará State, northern Brazil (continuation).

Non-Human Primate Taxa	Entamoebidae	Ciliophora	Parabasalia	*Giardia* *duodenalis*	*Blastocystis* sp.	Thin-Shelled Nematode Egg	Nematode Larvae	*Trichuris* sp.	Phylum Acanthocephala	Infraclass Trematoda	Total
Cebidae											
*Saimiri collinsi* (n = 30)	3 (10%)	-	23 (76.7%)	-	-	1 (33.3%)	1 (33.3%)	-	-	-	25 (83.3%) **
*Saimiri collinsi* and *Saimiri boliviensis* (n = 1)	-	-	1 (100%)	-	-	-	-	-	-	-	1 (100%)
*Cebus olivaceus* (n = 4)	1 (25%)	-	-	-	-	1 (25%)	-	-	-	-	1 (25%)
*Cebus albifrons* (n = 3)	-	-	-	-	-	1 (33.3%)	-	-	-	-	1 (33.3%)
*Cebus kaapori* (n = 1)	-	-	-	-	1 (100%)	-	-	-	-	-	1 (100%)
*Sapajus apella* (n = 12)	-	-	1 (8.3%)	-	-	7 (58.3%)	-	-	-	-	8 (66.7%)
*Sapajus apella* and *Sapajus libidinosus* (n = 2)	-	-	-	-	-	1 (50%)	-	-	-	-	1 (50%)
*Sapajus apella* and *Cebus albifrons* (n = 2)	-	-	1 (50%)	-	-	-	-	-	-	-	1 (50%)
*Sapajus apella, Sapajus libidinosus* and *Cebus albifrons* (n = 1)	1 (100%)	-	-	-	-	1 (100%)	-	-	-	-	1 (100%)
*Sapajus apella* and *Sapajus xanthosternos* (n = 3)	-	-	1 (33.3%)	-	-	-	-	-	-	-	1 (33.3%)
*Sapajus libidinosus* (n = 1)	-	1 (100%)	-	-	-	1 (100%)	-	-	-	-	1 (100%)
Pitheciidae											
*Cheracebus purinus* and *Plecturocebus hoffmannsi* (n = 1)	1 (100%)	-	-	-	-	-	-	-	-	-	1 (100%)
*Chiropotes utahickae* (n = 2)	-	-	-	-	-	1 (50%)	-	-	-	1 (50%)	2 (100%)
Atelidae											
*Alouatta caraya* (n = 25)	7 (28%)	-	6 (24%)	-	1 (4%)	1 (4%)	-	-	-	-	15 (60%)
Cercopithecidae											
*Chlorocebus aethiops* (n = 11)	7 (63.6%)	-	-	2 (18.2%)	1 (9.1%)	-	-	1 (9.1%)	-	-	7 (63.6%) **
Total (n = 206)	40 (19.4%)	3 (1.4%)	40 (19.4%)	3 (1.4%)	4 (1.9%)	20 (9.7%)	2 (1%)	1 (0.5%)	1 (0.5%)	2 (1%)	96 (46.6%)

**: Mixed infections; n: number of samples.

**Table 8:** Multivariate analysis based on logistic regression applied to variables addressed in animal management questionnaires associated with parasites detected in fecal samples collected from non-human primates kept in different Brazilian institutions, from March 2021 to June 2023.

Global Positivity Recorded for Gastrointestinal Parasites	Total	Odds Ratio	*p*-Value
Primates’ occupational density in each enclosure			
More than 1 animal (n = 309)	125 (40.4%)	1.74	0.01
1 animal (n = 150)	42 (28%)		
Use of animals for research protocol purposes			
Yes (n = 93)	48 (51.6%)	2.09	0.03
No (n = 366)	119 (32.5%)		
Quarantine			
Yes (n = 368)	119 (32.3%)	0.45	0.00
No (n = 91)	48 (52.7%)		
Parabasalia	Total	Odds Ratio	*p*-value
Use of animals for research protocol purposes			
Yes (n = 93)	29 (31.2%)	6.39	0.00
No (n = 366)	21 (5.7%)		
Thin-shelled nematode egg	Total	Odds Ratio	*p*-value
Food cleaning			
Water and chemicals (n = 430)	24 (5.6%)	0.14	0.01
Only water (n = 29)	6 (20.7%)		
Quarantine			
Yes (n = 368)	14 (3.8%)	0.14	0.00
No (n = 91)	16 (17.6%)		
Nematode Larvae	Total	Odds Ratio	*p*-value
Animal’s water source			
Treated water used for public supply (n = 73)	9 (12.3%)	5.13	0.00
Natural (n = 386)	8 (2.1%)		

n: number of samples.

**Table 9:** Multivariate analysis based on logistic regression applied to variables addressed in animal management questionnaires associated with parasites detected in fecal samples collected from non-human primates kept in different Brazilian institutions, from March 2021 to June 2023 (continuation).

Global Positivity Recorded for Gastrointestinal Parasites	Total	Odds Ratio	*p*-Value
Periodicity of antiparasitic drugs supplied to animals			
Once a year (n = 332)	93 (28%)	0.34	0.00
Not administered (n = 127)	74 (58.3%)		
Implementation of post-infection vacant period in enclosures			
Yes (n = 221)	45 (20.4%)	0.40	0.00
No (n = 238)	122 (51.3%)		
Entamoebidae	Total	Odds Ratio	*p*-value
Enclosures’ hygienization			
Dry and wet cleaning (n = 258)	49 (19%)	4.45	0.00
Only dry cleaning (n = 201)	8 (4%)		
Parabasalia	Total	Odds Ratio	*p*-value
Animals presenting gastrointestinal disorder symptoms in the previous six months			
No (n = 172)	26 (15.1%)	2.17	0.03
Yes (n = 287)	24 (8.4%)		
Thin-shelled nematode egg	Total	Odds Ratio	*p*-value
Periodicity of antiparasitic drugs supplied to animals			
Once a year (n = 332)	7 (2.1%)	0.12	0.00
Not administered (n = 127)	23 (18.1%)		
Phylum Acanthocephala	Total	Odds Ratio	*p*-value
Enclosures’ hygienization			
Dry and wet cleaning (n = 258)	1 (0.4%)	0.04	0.00
Only dry cleaning (n = 201)	16 (8%)		

n: number of samples.

**Table 10:** Correlation between variables addressed in questionnaires about the routine of handlers from Institutions A to E and coproparasitological results in the professionals’ samples.

Variables	Entamoebidae	*p*-Value
Sex		
Female (n = 22)	4 (18.2%)	0.74 ^a^
Male (n = 52)	8 (15.4%)	
Institution		
A (n = 20)	3 (15%)	0.04 *^b^
B (n = 5)	0	
C (n = 18)	1 (5.5%)	
D (n = 22)	8 (36.4%)	
E (n = 9)	0	
Occupation		
Only primate handler or primate and food handler, simultaneously (n = 70)	12 (17.1%)	1.00 ^a^
Only food handler (n = 4)	0	
Time working with non-human primates		
Up to 5 years (n = 41)	9 (21.9%)	0.20 ^a^
More than 5 years (n = 33)	3 (9.1%)	
Administration of antiparasitic drugs		
Yes (n = 27)	3 (11.1%)	0.35 ^a^
No (n = 44)	9 (20.4%)	
Do not remember (n = 3) *	-	
Last stool examination		
Never did (n = 13)	1 (7.7%)	0.53 ^b^
1 year ago (n = 6)	1 (16.5%)	
Between 1 and 5 years (n = 21)	6 (28.6%)	
Over 5 years ago (n = 14)	2 (14.3%)	
Do not remember (n = 20) *	-	
Diarrhea in the last 6 months		
Yes (n = 33)	7 (21.2%)	0.35 ^a^
No (n = 41)	5 (12.2%)	
Helminths ever found in fecal samples		
Yes (n = 25)	5 (20%)	0.52 ^a^
No (n = 49)	7 (14.3%)	

* Variables not included in the analysis; ^a^—Fisher’s exact test; ^b^—Chi-square test; *^b^: *p* < 0.05.

**Table 11:** Correlation between variables addressed in questionnaires about the routine of handlers from Institutions A to E and coproparasitological results in the professionals’ samples (continuation).

Variables	Entamoebidae	*p*-Value
Personal protective equipment used to handle non-human primates		
Pants, closed-toe shoes, half-sleeved blouse and rubber/leather glove (n = 12)	2 (16.7%)	0.97 ^b^
Pants, closed-toe shoes, long blouse/jalecoat/overalls and rubber/leather gloves (n = 19)	3 (15.8%)	
Pants, closed-toe shoes, half-sleeved blouse and procedure glove (n = 4)	1 (25%)	
Pants, closed-toe shoes, long blouse/jalecoat/overalls and procedure gloves (n = 18)	3 (16.7%)	
Pants, closed shoes, half-sleeved blouse and no gloves (n = 5)	0	
Pants, closed shoes, long blouse/jalecoat/overall, procedure glove and rubber/leather glove (n = 16)	3 (18.7%)	
Place where personal protective equipment hygienization is carried out		
Only at home or at home and work (n = 44)	4 (9.1%)	0.057 ^a^
Only at work (n = 30)	8 (26.7%)	
Activities performed right after enclosure hygienization		
Body cleaning (n = 21)	7 (33.3%)	0.09 ^b^
Hand/feet cleaning only (n = 37)	4 (10.8%)	
Activities not associated with cleaning (n = 11)	1 (9.1%)	
Do not clean the enclosures (n = 5) *	-	
Knowledge about the zoonotic transmission of gastrointestinal parasites		
Yes (n = 66)	10 (15.1%)	0.60 ^a^
No (n = 8)	2 (25%)	

* Variables not included in the analysis; ^a^—Fisher’s exact test; ^b^—Chi-square test.

## Data Availability

Data on New World, Old World, Great Ape and Prosimian NHP taxa, as well as on the number of enclosures, assessed individuals and collected samples, are shown in Supplementary Table S1. Also, all the variables analyzed via univariate analysis are available in Supplementary Table S2.

## Supplementary Materials

The following supporting information can be downloaded at: https://www.mdpi.com/article/10.3390/pathogens12121410/s1, Table S1: Non-human primate herd, number of enclosures and samples collected from Institutions (A to E) in different regions of Brazil from March 2021 to June 2023; Table S2: Univariate analysis of variables from animal management questionnaires associated with parasites detected in fecal samples from non-human primates in different Institutions in Brazil from March 2021 to June 2023.

## References

[1] Estrada A., Garber P.A., Rylands A.B., Roos C., Fernandez-Duque E., Di Fiore A., Nekaris K.A.-I., Nijman V., Heymann E.W., Lambert J.E. (2017). Impending Extinction Crisis of the World’s Primates: Why Primates Matter. Sci. Adv..

[2] Johnson-Delaney C.A. (2009). Parasites of Captive Nonhuman Primates. Vet. Clin. N. Am. Exot. Anim. Pract..

[3] Malan F.S., Horak I.G., de Vos V., van Wyk J.A. (1997). Wildlife Parasites: Lessons for Parasite Control in Livestock. Vet. Parasitol..

[4] Michaud C., Tantalean M., Ique C., Montoya E., Gozalo A. (2003). A Survey for Helminth Parasites in Feral New World Non-Human Primate Populations and Its Comparison with Parasitological Data from Man in the Region. J. Med. Primatol..

[5] Barbosa A.d.S., Pissinatti A., Dib L.V., de Siqueira M.P., Cardozo M.L., Fonseca A.B.M., de Barros Oliveira A., da Silva F.A., Uchôa C.M.A., Bastos O.M.P. (2015). *Balantidium coli* and Other Gastrointestinal Parasites in Captives Non-Human Primates of the Rio de Janeiro, Brazil. J. Med. Primatol..

[6] Levecke B., Dorny P., Geurden T., Vercammen F., Vercruysse J. (2007). Gastrointestinal Protozoa in Non-Human Primates of Four Zoological Gardens in Belgium. Vet. Parasitol..

[7] Rylands A.B., Valladares-Pádua C., Silva R.d.R., Catão-Dias J.L., Pissinatti A., Guimarães M.A.d.B.V., Fowler M.E. (2008). Order Primates (Primates). Biology, Medicine, and Surgery of South American Wild Animals.

[8] Schuster F.L., Ramirez-Avila L. (2008). Current World Status of *Balantidium coli*. Clin. Microbiol. Rev..

[9] Pérez Cordón G., Hitos Prados A., Romero D., Sánchez Moreno M., Pontes A., Osuna A., Rosales M.J. (2008). Intestinal Parasitism in the Animals of the Zoological Garden “Peña Escrita” (Almuñecar, Spain). Vet. Parasitol..

[10] Regan C.S., Yon L., Hossain M., Elsheikha H.M. (2014). Prevalence of *Entamoeba* Species in Captive Primates in Zoological Gardens in the UK. PeerJ.

[11] Verweij J.J., Vermeer J., Brienen E.A.T., Blotkamp C., Laeijendecker D., van Lieshout L., Polderman A.M. (2003). *Entamoeba histolytica* Infections in Captive Primates. Parasitol. Res..

[12] Köster P.C., Dashti A., Bailo B., Muadica A.S., Maloney J.G., Santín M., Chicharro C., Migueláñez S., Nieto F.J., Cano-Terriza D. (2021). Occurrence and Genetic Diversity of Protist Parasites in Captive Non-Human Primates, Zookeepers, and Free-Living Sympatric Rats in the Córdoba Zoo Conservation Centre, Southern Spain. Animals.

[13] Barbosa A.d.S., Pinheiro J.L., dos Santos C.R., de Lima C.S.C.C., Dib L.V., Echarte G.V., Augusto A.M., Bastos A.C.M.P., Antunes Uchôa C.M., Bastos O.M.P. (2020). Gastrointestinal Parasites in Captive Animals at the Rio de Janeiro Zoo. Acta Parasitol..

[14] Figueiredo M.A.P., Manrique W.G., Nogueira R.M.S. (2018). Survey of Gastrointestinal Parasites of the Center for Screening of Wild Animals from São Luís, Maranhão State, Brazil. Ars Vet..

[15] Batista A.I.V., de Lucena G.V.C., Nery T.F.L., Batista C.C.N., Batista J.S., Winkeler I.E., Rolim C.M.d.M., Coelho W.A.C., Rocha E.L.B., Lima V.F.S. (2021). Gastrointestinal Parasites in Wild and Exotic Animals from a Zoobotanical Park in Northeast of Brazil. Res. Soc. Dev..

[16] Macedo E.C., Teixeira R.E.R., Santos S.P., Oliveira V.d.J., de Antonio E.S., Prado I.S., Aguiar C.d.S., Tomazi L., da Silva M.B., Fraga R.E. (2022). Gastrointestinal Parasites in Non-Human Primates from the Wildlife Animal Screening Center of Vitória Da Conquista, Bahia, Brazil. Res. Soc. Dev..

[17] dos Santos I.G., Batista A.I.V., da Silva W.S.I., Oliveira Neto M.B., Schettino S.C., Oliveira M.R., Ramos R.A.N., Alves L.C., Bezerra-Santos M., Lima V.F.S. (2022). Gastrointestinal Parasites in Captive Wild Animals from Two Brazilian Zoological Gardens. Res. Soc. Dev..

[18] Li M., Zhao B., Li B., Wang Q., Niu L., Deng J., Gu X., Peng X., Wang T., Yang G. (2015). Prevalence of Gastrointestinal Parasites in Captive Non-human Primates of Twenty-four Zoological Gardens in China. J. Med. Primatol..

[19] Vonfeld I., Prenant T., Polack B., Guillot J., Quintard B. (2022). Gastrointestinal Parasites in Non-Human Primates in Zoological Institutions in France. Parasite.

[20] Martin-Solano S., Carrillo-Bilbao G.A., Ramirez W., Celi-Erazo M., Huynen M.-C., Levecke B., Benitez-Ortiz W., Losson B. (2017). Gastrointestinal Parasites in Captive and Free-Ranging *Cebus albifrons* in the Western Amazon, Ecuador. Int. J. Parasitol. Parasites Wildl..

[21] Mati V.L.T., Junior F.C.F., Pinto H.A., De Melo A.L. (2013). *Strongyloides Cebus* (Nematoda: Strongyloididae) in *Lagothrix cana* (Primates: Atelidae) from the Brazilian Amazon: Aspects of Clinical Presentation, Anatomopathology, Treatment, and Parasitic Biology. J. Parasitol..

[22] Calle P.P., Ott Joslin J. (2015). New World and Old World Monkeys. Fowler’s Zoo and Wild Animal Medicine.

[23] Barbosa A.D.S., Dib L.V., Uchôa C.M.A., Bastos O.M.P., Pissinatti A. (2017). *Trypanoxyuris* (*Trypanoxyuris*) *minutus* (Schneider, 1866) among *Alouatta guariba clamitans* (Cabrera, 1940) in the State of Rio de Janeiro, Brazil. J. Med. Primatol..

[24] Falla A.C., Brieva C., Bloor P. (2015). Mitochondrial DNA Diversity in the Acanthocephalan *Prosthenorchis elegans* in Colombia Based on Cytochrome c Oxidase I (COI) Gene Sequence. Int. J. Parasitol. Parasites Wildl..

[25] da Silva M.B., de Oliveira D.F., Santos F.V., Aguiar C.d.S., Prado I.S., Brandão D.A., Pereira A.A., de Queiroz T.S., Tomazi L., Fraga R.E. (2022). Gastrointestinal Parasites in Wild and Exotic Animals from a Zoo in the State of Bahia, Brazil—First Record. Res. Soc. Dev..

[26] da Silva A.S., Coradini G.P., Gressler L.T., Soares J.F., Lara V.M., Carregaro A.B., Monteiro S.G. (2008). Ocorrência de Protozoários Gastrintestinais em Primatas Mantidos em Cativeiro na Região Sul do Brasil. Cienc. Rural.

[27] Sprenger L.K., Yoshitani U.Y., Buzatti A., Molento M.B. (2018). Occurrence of Gastrointestinal Parasites in Wild Animals in State of Paraná, Brazil. An. Acad. Bras. Ciênc..

[28] da Silva M.T.F., Aquim E.C., De Sousa T.P.R., Gomes N.R.d.S., De Vasconcelos A.R.O., Andrade Silveira C.C.d.O., Junior Costa F.M., Evangelista L.S.d.M. (2018). Occurrence of Intestinal Parasites in *Alouatta caraya* of the Zoobotanical Park of Teresina, Piauí, Brazil. Acta Sci. Vet..

[29] Mewius A., Lusa E.R., Pertille J.G., Reis T.D.D., Pletsch J.A., França R.T., de Castro L.L.D. (2021). Endoparasites in Group of Wild Animals Raised in Captivity. Pesq. Vet. Bras..

[30] Faust E.C., D’Antoni J.S., Odom V., Miller M.J., Peres C., Sawitz W., Thomen L.F., Tobie J., Walker J.H. (1938). A Critical Study of Clinical Laboratory Technics for the Diagnosis of Protozoan Cysts and Helminth Eggs in Feces. Am. J. Trop. Med. Hyg..

[31] Ritchie L.S. (1948). An Ether Sedimentation Technique for Routine Stool Examinations. Bull US Army Med. Dep..

[32] Young K.H., Bullock S.L., Melvin D.M., Spruill C.L. (1979). Ethyl Acetate as a Substitute for Diethyl Ether in the Formalin-Ether Sedimentation Technique. J. Clin. Microbiol..

[33] Lutz A. (1919). *Schistosomum mansoni* and Schistosomatosis Observed in Brazil. Mem. Inst. Oswaldo Cruz.

[34] Cringoli G., Maurelli M.P., Levecke B., Bosco A., Vercruysse J., Utzinger J., Rinaldi L. (2017). The Mini-FLOTAC Technique for the Diagnosis of Helminth and Protozoan Infections in Humans and Animals. Nat. Protoc..

[35] Munene E., Otsyula M., Mbaabu D.A.N., Mutahi W.T., Muriuki S.M.K., Muchemi G.M. (1998). Helminth and Protozoan Gastrointestinal Tract Parasites in Captive and Wild-Trapped African Non-Human Primates. Vet. Parasitol..

[36] Khatun M.M., Begum N., Mamun M.A.A., Mondal M.M.H., Shakif-Ul-Azam M. (2014). Coprological Study of Gastrointestinal Parasites of Captive Animals at Rangpur Recreational Garden and Zoo in Bangladesh. J. Threat. Taxa.

[37] Dawet A., Yakubu D.P., Butu H.M. (2013). Survey of Gastrointestinal Parasites of Non-Human Primates in Jos Zoological Garden. J. Primatol..

[38] Genoy-Puerto A., Santos R.C.F., Guimarães-Luiz T., Sánchez-Sarmiento A.M., Zacariotti R.L., Catão-Dias J.L., Matushima E.R. (2016). Epizootic Amebiasis Outbreak in Wild Black Howler Monkeys (*Alouatta caraya*) in a Wildlife Facility during Captivity Prior to Translocation—Recommendations to Wildlife Management Programs. Braz. J. Vet. Res. Anim. Sci..

[39] Elsheikha H.M., Regan C.S., Clark C.G. (2018). Novel *Entamoeba* Findings in Nonhuman Primates. Trends Parasitol..

[40] Toft J.D. (1982). The Pathoparasitology of the Alimentary Tract and Pancreas of Nonhuman Primates: A Review. Vet. Pathol..

[41] Ulrich R., Böer M., Herder V., Spitzbarth I., Hewicker-Trautwein M., Baumgärtner W., Wohlsein P. (2010). Epizootic Fatal Amebiasis in an Outdoor Group of Old World Monkeys: *Entamoeba histolytica* in Colobinae. J. Med. Primatol..

[42] Souza C.S., Bandeira L.L.B., Mariano A.K.N.O., Nunes M.d.P.S.F., Neto J.D.d.S. (2019). Amebíase no contexto da emergência: Análise do perfil de internações e morbimortalidade nos Estados brasileiros em 5 anos. Rev. Soc. Bras. Clin. Med..

[43] Santos F.L.N., Soares N.M. (2008). Mecanismos fisiopatogênicos e diagnóstico laboratorial da infecção causada pela *Entamoeba histolytica*. J. Bras. Patol. Med. Lab..

[44] Köster P.C., Martínez-Nevado E., González A., Abelló-Poveda M.T., Fernández-Bellon H., de la Riva-Fraga M., Marquet B., Guéry J.-P., Knauf-Witzens T., Weigold A. (2022). Intestinal Protists in Captive Non-Human Primates and Their Handlers in Six European Zoological Gardens. Molecular Evidence of Zoonotic Transmission. Front. Vet. Sci..

[45] Stensvold C.R., Arendrup M.C., Jespersgaard C., Mølbak K., Nielsen H.V. (2007). Detecting *Blastocystis* Using Parasitologic and DNA-Based Methods: A Comparative Study. Diagn. Microbiol. Infect. Dis..

[46] Hinde K. (2007). Milk Composition Varies in Relation to the Presence and Abundance of *Balantidium coli* in the Mother in Captive Rhesus Macaques (*Macaca mulatta*). Am. J. Primatol..

[47] Kim J.C.S., Abee C.R., Wolf R.H. (1978). Balantidiosis in a Chimpanzee (*Pan Troglodytes*). Lab. Anim..

[48] Lankester F., Mätz-Rensing K., Kiyang J., Jensen S.A., Weiss S., Leendertz F.H. (2008). Fatal Ulcerative Colitis in a Western Lowland Gorilla (*Gorilla gorilla gorilla*). J. Med. Primatol..

[49] Al-Tayib O., Abdoun K.A. (2013). *Balantidium coli* infection in hamadryas baboon (*Papio hamadryas*) in Saudi Arabia: A case report. J. Anim. Plant Sci..

[50] Kalishman J., Paul-Murphy J., Scheffler J., Thomson J.A. (1996). Survey of *Cryptosporidium* and *Giardia* spp. in a Captive Population of Common Marmosets. Lab. Anim. Sci..

[51] Santos C.S., de Jesus V.L.T., McIntosh D., Carreiro C.C., Batista L.C.O., do Bomfim Lopes B., Neves D.M., Lopes C.W.G. (2017). Morphological, Ultrastructural, and Molecular Characterization of Intestinal Tetratrichomonads Isolated from Non-Human Primates in Southeastern Brazil. Parasitol. Res..

[52] Hamlen H.J., Lawrence J.M. (1994). Giardiasis in Laboratory-Housed Squirrel Monkeys: A Retrospective Study. Lab. Anim. Sci..

[53] Little M.D. (1981). Differentiation of Nematode Larvae in Coprocultures: Guidelines for Routine Practice in Medical Laboratories. WHO Tech. Rep. Ser..

[54] Guerrero M.F., Serrano-Martínez E., Tantaleán V.M., Quispe H.M., Casas V.G. (2012). Identificación de parásitos gastrointestinales en primates no humanos del Zoológico Parque Natural de Pucallpa, Perú. Rev. Investig. Vet. Perú.

[55] Martins A.V., Corrêa L.L., Ribeiro M.S., Lobão L.F., Dib L.V., Palmer J.P.S., De Moura L.C., Knackfuss F.B., Uchôa C.M.A., Molento M.B. (2023). Prevalence, Risk Factors and Diagnosis of Helminths in Thoroughbred Horses Kept at Training Centers in Rio de Janeiro, Brazil. J. Equine Vet. Sci..

[56] Dib L.V., Pissinatti A., Moreira S.B., Amendoeira M.R.R., Barbosa A.d.S. (2022). Infection by *Prosthernorchis elegans* (Diesing, 1851) in Captive *Callithrix aurita* (É. Geoffroy, 1812) and *Leontopithecus rosalia* (Linnaeus, 1766) from Rio de Janeiro, Brazil. J. Med. Primatol..

[57] Zárate-Ramos J., Gómez-Garza M.A., Rodríguez-Tovar L.E., Escareño J.H., Contreras-Lozano J.A. (2018). An Alternative Treatment Against Acanthocephala (*Prosthenorchis elegans*) in Captive Squirrel Monkeys (*Saimiri sciureus*) in Mexico. J. Parasitol..

[58] Sricharern W., Inpankaew T., Kaewmongkol S., Jarudecha T., Inthong N. (2021). Molecular Identification of *Trichuris trichiura* and *Hymenolepis diminuta* in Long-Tailed Macaques (*Macaca fascicularis*) in Lopburi, Thailand. Vet. World.

[59] Pereira W.L.A., Conga D.M.F., Da Silva K.S.M., Da Silva R.J., Imbeloni A.A. (2021). Anatomopathological Lesions of Infection Caused by *Platynosomum illiciens* (Braun, 1901) in Neotropical Primates Kept in Captivity. J. Med. Primatol..

[60] da Silva L.C.S., Silva K.S.M., Monteiro F.O.B., Muniz J.A.P.C., Castro P.H.G. (2012). Platinosomíase em cuxiú (*Chiropotes satanas utahicki*). Ciênc. Vet. Trop..

[61] do Carmo A.M., Salgado C.A. (2003). Ocorrência de Parasitos Intestinais Em *Callithrix* sp. (Mammalia, Primates, Callithrichidae). Rev. Bras. Zoociências.

[62] Maritz J.M., Land K.M., Carlton J.M., Hirt R.P. (2014). What Is the Importance of Zoonotic Trichomonads for Human Health?. Trends Parasitol..

