# Supplementary material for: Gastrointestinal Parasites Affecting Non-Human Primates That Are Kept Ex Situ and Their Handlers in Different Brazilian Institutions: Diagnosis and Analysis of Risk Factors
Source: Pathogens. 2023 Nov 30;12(12):1410. doi: 10.3390/pathogens12121410 (PMC10745941; doi:10.3390/pathogens12121410)
Supplement: Supplementary file 1 [file pathogens-12-01410-s001.zip › Table S1.pdf]

Suplementary material Table S1: Non-human primate herd, number of enclosures and samples collected from Institutions (A to E) in different regions of Brazil from March 2021 to June 2023

Non-human Primates	Number of enclosures	Total number of animals	Samples collected
**Institution A**			
**Family Callitrichidae**			
Callithrix jacchus	1	3	1
Callithrix penicillata	2	4	2
Mico melanurus	1	3	1
Saguinus bicolor	2	4	2
Saguinus fuscicollis	1	4	1
Saguinus midas	1	3	1
Saguinus niger	1	2	1
Leontopithecus chrysomelas	1	5	1
Leontopithecus rosalia	1	1	1
**Family Aotidae**			
Aotus trivirgatus	3	4	3
**Family Cebidae**			
Sapajus nigritus	1	2	1
Sapajus nigritus [1]	1	1	-
Cebus olivaceus [1]	1	5	1
**Family Pitheciidae**			
Plecturocebus vieirai	3	6	3
Chiropotes utahickae [1]	1	1	-
**Family Atelidae**			
Alouatta caraya	2	12	2
Alouatta guariba	1	6	1
Alouatta sp.*	-	-	20
Ateles chamek	3	5	3
*Ateles marginatus***	3	12	6
Brachyteles arachnoides	1	3	1
**Family Cercopithecidae**			
Mandrillus sphinx	1	1	1
Papio hamadryas	4	4	4
**Family Hominidae**			
*Pan troglodytes***	1	2	8
**Family Lemuridae**			
*Lemur catta***	1	4	3

Non-human Primates	Number of enclosures	Total number of animals	Samples collected
**Institution B**			
**Family Callitrichidae**			
*Callithrix sp.**	-	-	5
Callithrix penicillata	15	29	15
**Family Cebidae**			
*Sapajus sp.**	-	-	2
Sapajus libidinosus	14	30	14
**Institution C**			
**Family Callitrichidae**			
*Callithrix aurita**/****	8	25	16
Callithrix geoffroyi	1	2	1
Hybrid *Callithrix*	1	1	1
Cebuella pygmaea	2	4	2
Mico chrysoleucus	1	4	1
Mico mauesi [2]	1	1	1
Saguinus bicolor [2]	1	1	-
Saguinus bicolor [3]	1	1	1
*Saguinus bicolor***	4	7	5
Saguinus martinsi	2	2	2
Saguinus midas	3	13	3
Saguinus niger [3]	1	1	-
*Leontopithecus chrysomelas**/****	85	201	93
Leontopithecus chrysopygus	4	6	4
*Leontopithecus rosalia**/****	6	27	8
Hybrid *Leontopithecus*	1	1	1
**Family Aotidae**			
Aotus sp.	2	2	2
Aotus nigriceps	1	1	1
**Family Cebidae**			
Sapajus apella	1	1	1
*Sapajus robustus****	1	1	3
Sapajus xanthosternos	12	41	12
**Family Atelidae**			
Alouatta discolor	1	1	1
Alouatta caraya	1	1	1

*Alouatta guariba****	4	12	7
Ateles paniscus	1	1	1
**Non-human Primates**	**Number of enclosures**	**Total number of animals**	**Samples collected**
**Institution C**			
**Family Pitheciidae**			
Callicebus melanochir	1	1	1
Plecturocebus caquetensis	1	1	1
Plecturocebus dubius	1	1	1
Plecturocebus vieirai	1	1	1
Pithecia mittermeieri	1	2	1
Pithecia monachus	1	2	1
Chiropotes satanás [4]	1	1	-
Chiropotes satanas	1	1	1
Cacajao melanocephalus [4]	1	1	1
**Institution D**			
**Family Callitrichidae**			
Callithrix penicillata	2	4	2
*Callithrix jacchus***	5	9	6
*C. jacchus x C. penicillata***	12	40	14
Mico humeralifer [5]	1	1	1
Mico argentatus	1	4	1
Saguinus midas [6]	1	2	1
Saguinus bicolor	3	5	3
Saguinus ursulus	8	8	8
*Leontocebus weddelli***	9	19	11
Leontopithecus chrysomelas	5	14	5
Leontopithecus chrysomelas [5]	1	1	-
*Callimico goeldii***	3	8	4
**Family Aotidae**			
Aotus infulatus	48	100	48
**Family Cebidae**			
Sapajus apella	12	64	12
Sapajus apella [7]	2	3	2
Sapajus apella [8]	2	8	2
Sapajus apella [9]	1	4	1
Sapajus apella [10]	1	2	1

Non-human Primates	Number of enclosures	Total number of animals	Samples collected
**Institution D**			
**Family Cebidae**			
*Saimiri collinsi***	29	175	30
Saimiri collinsi [12]	1	1	1
Saimiri boliviensis [12]	1	1	-
Cebus olivaceus	4	13	4
Cebus albifrons	3	10	3
Cebus albifrons [8]	2	7	-
Cebus albifrons [10]	1	1	-
Cebus kaapori	1	2	1
Sapajus apella [11]	3	10	3
Sapajus xanthosternos [11]	3	5	-
Sapajus libidinosus	1	10	1
Sapajus libidinosus [7]	2	5	-
Sapajus libidinosus [9]	1	1	-
Sapajus libidinosus [10]	1	1	-
Sapajus nigritus [9]	1	1	-
**Family Pitheciidae**			
Callicebus sp. [6]	1	2	-
Plecturocebus hoffmannsi [13]	1	1	-
Cheracebus purinus [13]	1	1	1
Chiropotes utahickae	2	8	2
**Family Atelidae**			
*Alouatta caraya***	14	46	25
Ateles paniscus	1	5	1
Ateles marginatus	1	2	1
**Family Cercopithecidae**			
*Chlorocebus aethiops***	10	53	11
**Institution E**			
**Family Atelidae**			
*Alouatta guariba***	24	41	25
**Total:**	**511**		

*: Free-living animals at the Institution

** request for collection of more fecal samples from non-human primates by those accounting for providing care to the animals

*** request for collection of fecal samples from animals who had recently arrived at the Institutions

/* request for collection of fecal samples from animals who had recently arrived at the Institutions and from hospitalized animals

Note: Animals with the same superscript number next to them were sharing the same enclosure.
